# Improving detection of low-abundant pharmaceuticals and neurotransmitters via quadrupole isolation in MALDI mass spectrometry imaging

**DOI:** 10.1007/s00216-026-06659-z

**Published:** 2026-07-14

**Authors:** Karina A. Vargas, Sarah Diez, Michael Becker, Elizabeth K. Neumann

**Affiliations:** 1 Department of Chemistry, University of California, Davis, Davis, USA; 2 Boehringer Ingelheim Pharma GmbH, Biberach an der Riss, Germany

**Keywords:** MALDI mass spectrometry imaging, MS/MS, Neurotransmitters, Quadrupole isolation, Small molecules

## Abstract

Matrix-assisted laser desorption/ionization mass spectrometry imaging (MALDI MSI) is a powerful tool for mapping the spatial distribution of pharmaceuticals and metabolites in tissue. However, its effectiveness in the low-mass range (< 400 *m*/*z*) is impacted by matrix interference and ion suppression, reducing signal-to-noise (S/N) ratios. To address this challenge, we assessed a targeted quadrupole isolation strategy using a MALDI q-TOF mass spectrometer. This approach selectively isolates narrow windows around individual analytes *m*/*z* to reduce background noise and enhance S/N, therefore generating a smaller selective ion packet to the TOF analyzer. We applied this method to detect acetaminophen, paracetamol sulfate, caffeine, paraxanthine, as well as dopamine and its metabolites (HVA, 3-MT, and DOPAC) within murine liver and brain tissue sections, respectively. We utilized a range of isolation windows (e.g., 1, 5, 10, 20, 100 *m*/*z*) around each target *m*/*z* value. This dynamic isolation significantly decreased chemical background without fragmenting the target ions. As a result, we achieved precise spatial localization of parent drugs and their metabolites. Compared to untargeted acquisition, this method improved S/N by over 50% for all analytes within ≤10 *m*/*z* isolation ranges, while preserving tissue morphology. This targeted isolation approach extends the capabilities of MALDI MSI and offers a robust and scalable solution for analyzing low-mass xenobiotics and metabolites in situ.

**Graphical abstract:**

**Figure:**
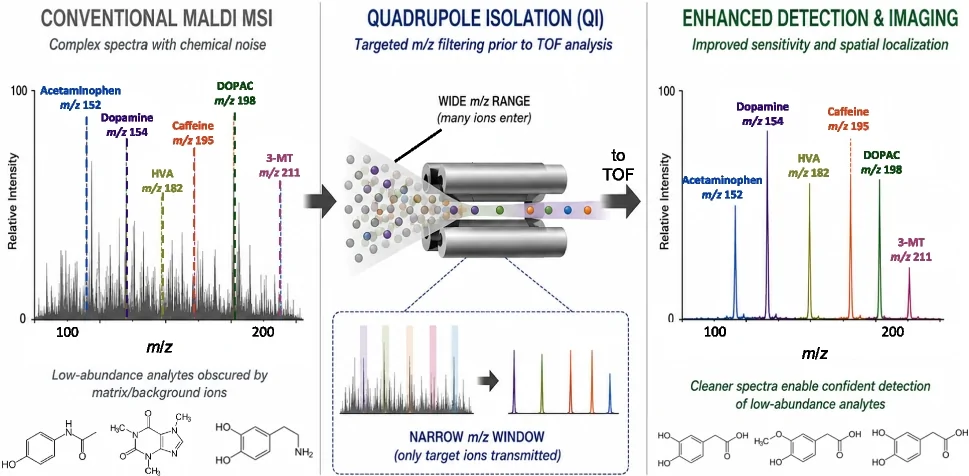

**Supplementary Information:**

The online version contains supplementary material available at https://doi.org/10.1007/s00216-026-06659-z.

## Introduction

Understanding the abundance and distribution of drugs and their metabolites within tissue is a fundamental challenge in drug development. This requires sensitive methods capable of detecting low-abundant small molecules, even as they are transported and metabolized within complex tissue microenvironments [1]. Drug distribution is inherently heterogeneous [2] as compounds often accumulate unevenly across organs, cell types, and tissue compartments [3]. This is due to both physiochemical and physiological properties of the drugs [4]. Without spatially resolved information, critical phenomena like off-target site accumulation [5] or localized toxicity may go undetected [6]. Spatially resolved techniques like matrix-assisted laser desorption/ionization mass spectrometry imaging (MALDI MSI) preserve tissue morphology and enable direct correlation between molecular signals and anatomical structures [7]. MALDI MSI enables label-free, direct detection of drug distributions and metabolic transformations within intact tissue sections [5, 8, 9]. MALDI MSI involves coating a tissue with a UV-absorbing chemical matrix which preserves spatial information of small molecules and facilitates desorption and ionization [10]. A laser is rastered across the tissue, generating a mass spectrum at each imaging position, or pixel [11]. MSI methods not only distinguish parent compounds and endogenous molecules from their metabolites [12], but also reveal their precise localization and resulting metabolites within tissues [13, 14]. Such spatial resolution is essential to confirm drug delivery at the intended active sites and to identify accumulation in non-target organs that may contribute to toxicity [15]. This detailed insight is critical for accurately characterizing drug behavior and effects within complex biological systems. Beyond mapping drug and metabolite localization, these approaches can simultaneously provide pharmacodynamic and biomarker information, offering a comprehensive view that is invaluable at multiple stages of drug development [8].

Small molecules within biological tissues frequently experience ion suppression during the MALDI ionization process or become obscured within highly complex spectra due to their inherent low abundance [16]. This can result in reduced S/N ratios and limited detectability. These conditions limit detectability and complicate confident identification, even when ionization efficiency is adequate. While matrix-related chemical noise and spectral congestion can obscure low-mass analytes, additional constraints arise from the large number of co-generated ions that are transmitted into the mass analyzer, reducing effective utilization of the available ion packet by target species. In contrast to spatial analysis methods, traditional LC-MS-based bulk quantification methods reduce spectral complexity through chromatographic separation prior to ionization [17–20]. LC-MS analysis provides quantitation of drugs, but averages the signal across tissue samples, preventing spatial analysis and disrupting native tissue architecture and biochemical context [21], unless techniques like laser capture microdissection are utilized [22, 23]. Removing the spatial information may mask tissue regional differences and can lead to misinterpretation of pharmacological, pharmacodynamic, or toxicological effects [24]. Spatially tracking the localization of metabolites overcomes the difficulty of associating bulk metabolite analysis with a particular tissue or organ [25]. On-tissue chemical derivatization (OTCD) is a method compatible with spatial analysis that is commonly used as a precursor step for MALDI MSI to target many small-molecule drugs within tissue and overcome the S/N limitations, but there are molecules that do not have derivatization methods available [17, 27–29]. This attaches an easily ionizable functional group on the drug of interest to improve ionization efficiency and S/N [26]. However, OTCD sample preparation steps introduce variables such as solvent choices, concentration, and tissue immersion times that can reduce the ionization efficiency of analytes [29]. OTCD also introduces the possibility of post-source and in-source decay of analytes of interest [30–33]. An alternative to this is MALDI-2 MSI, where a second laser is added for higher energy laser-induced post-ionization step [31, 34] that increases low-abundant molecules S/N by two to three orders of magnitude [31–33, 35]. The ability of MALDI MSI to detect trace levels of unlabeled small molecules spatially provides critical insight into tissue-specific drug distribution and metabolism.

An orthogonal strategy for improving sensitivity is to reduce the number of irrelevant ions transmitted to the mass analyzer post-ionization. In hybrid instruments, excessive co-transmitted ions contribute to space-charge effects within injected ion packets, which can broaden peaks, degrade mass accuracy, and limit quantitative performance. Approaches such as continuous accumulation of selected ions (CASI) with Fourier-transform ion cyclotron resonance (FT-ICR) MS have demonstrated that restricting ion populations to selected *m*/z ranges can substantially improve detectability of low-abundance analytes [36–40]. Numerous studies have demonstrated the effectiveness of MALDI FT-ICR CASI for enhancing the detectability of low-abundance ions by selectively accumulating targeted *m*/*z*values over extended acquisition times, thereby reducing space-charge effects and improving ion utilization prior to transmission to the analyzer [36, 41–44]. Inspired by this concept, our goal is to increase S/N within the lower *m*/*z* range by applying quadrupole isolation to reduce ion population complexity prior to TOF analysis. In this study, we present a targeted approach to enhance the S/N ratio of low-abundance, low-mass drug compounds and neurotransmitters using MALDI MSI with applied quadrupole isolations. Our method leverages variable isolation widths within the quadrupole mass filter on the MALDI timsTOF fleX [45] to selectively transmit analytes of interest without inducing fragmentation, contrast to MS/MS. In a Q-TOF instrument, many co-transmitted, non-target ions can occupy a substantial fraction of the ion packet injected into the TOF analyzer. High ion populations within an analyzer lead to space-charge effects, reducing dynamic range, broadening peaks, and limiting sensitivity [46]. By restricting the transmitted ion population to a narrow *m*/*z *window using quadrupole isolation prior to TOF analysis, the number of irrelevant ions is reduced, thereby lowering space-charge effects and improving effective ion utilization [47]. This enhances S/N and specificity for targeted analytes, analogous to selected ion monitoring strategies that limit ion populations to defined *m*/*z *ranges for improved sensitivity and quantitation [48, 49]. This targeted filtering strategy is particularly effective within the lower mass range, where small molecules may otherwise be obscured by chemical noise (e.g., fragmentation of larger molecules and matrix peaks). To demonstrate the utility of this approach, we implement a dynamic isolation method optimized for the detection of exogenous, commonly used pharmaceuticals (e.g., acetaminophen and caffeine) and their associated metabolites, along with endogenous neurotransmitters. This capability is relevant for enhancing detection sensitivity in complex biological matrices and supporting early-stage drug development and toxicity screening. However, this method can be applied to both small and large molecules, including metabolites, peptides, hormones, and xenobiotics, enabling sensitive spatial mapping in pharmacology, metabolomics, environmental exposure, and biomarker studies.

## Experimental

### Chemicals and materials

All chemicals were purchased from Sigma-Aldrich (St. Louis, MO) without additional purification unless otherwise specified. FMP-10 reactive matrix was obtained from Tag-On (Uppsala, Sweden). Standards for dopamine metabolites homovanillic acid (HVA), 3-methoxytyramine (3-MT), and dihydroxyphenylacetic acid (DOPAC) were obtained from Merck (Darmstadt, Germany). Murine liver tissue was stored at −80 °C prior to cryosectioning to a 10-µm thickness using a cryostat (Leica Biosystems, Wetzlar, Germany) at −21 °C. Similarly, rat brain tissue was sectioned at a thickness of 12 µm at the level of the striatum (bregma levels 0.60–0.24 mm) using a cryostat set to −12 °C to −14 °C. All animal procedures were approved by either Boehringer Ingelheim (rat brain) or The University of California, Davis (mouse liver) institutional animal care and use committee (IACUC) and were conducted in accordance with applicable national and institutional guidelines for the care and use of laboratory animals. Brain from rat and liver from mouse tissues were extracted and immediately frozen.

### Small-molecule drug and metabolite solution and dopamine metabolite solution preparation

Caffeine, paraxanthine, paracetamol sulfate, and acetaminophen single stock mixture was prepared to approximately 1 mmol/L. Dilution series were carried out to create 0.05, 0.03, 0.02, 0.015, 0.01, 0.0075, 0.005, 0.003, 0.0025, 0.001, 0.0005, and 0.0001 mmol/L, respectively. Dilution series utilized 50:50 methanol/ultrapure water as solvent. Similarly, HVA, 3-MT, and DOPAC stock solutions were prepared to generate solutions with the same final concentrations as the previous small molecules. In this case, the dilution series utilized 30% methanol/miliQ H_2_O as the solvent. Stock and dilution series were stored in 4 °C for preservation.

### Dried droplet imaging sampling preparation

Tissue was thaw-mounted on indium tin oxide (ITO)–coated glass slides at 37 °C. One microliter of drug and metabolite solution was pipetted onto each liver section, while 0.5 µL of HVA/3-MT/DOPAC solution was used for each brain section, followed by matrix application. The volume was administered via calibrated microliter pipettes.

Dilution series of both drug and metabolite mixtures were spotted (1 µL per drop) on ITO slides, except for dopamine metabolites, where only 0.5 µL was used, followed by matrix application.

### Matrix application

Slides were sprayed with 40 mg/mL of 2,5-dihydroxybenzoicacid (DHB, Thermo Fisher Scientific, Waltham, MA) in 70% methanol using an HTX TM M3 Sprayer (HTX technologies, LLC, Chapel Hill, NC). Select spraying parameters include a flow rate of 120 µL/min, 1200 mm/min, 8 total passes, 2.5 mm track spacing, and a 50 °C nozzle temperature. Brain sections were spray-coated with FMP-10 (C_20_H_14_FN^+^) reactive matrix (10 mg FMP-10 dissolved in 70% ACN) using an automated HTX TM M3 sprayer following the protocol by Shariatgorji [50]. In brief, the matrix was applied with a flow rate of 80 µL/min, 1100 mm/min, 20 total passes, gas pressure of 6 p.s.i, and a 70 °C nozzle temperature.

### MALDI tims QTOF MSI

MALDI MSI was performed on the DHB matrix-sprayed slides less than 1 day after matrix application. All experiments were conducted using a MALDI timsTOF fleX mass spectrometer (Bruker Corp., Billerica, MA). The runs were controlled by timsControl (Bruker Corp, version 6.0) and flexImaging (Bruker Corp, version 7.5). Key imaging parameters for the dried droplet and liver tissue slides include mass scan range of *m*/*z* 50–1000, 50 µM laser raster width, and 100-pixel imaging area per each 1 µL spot. Collision energy was set to 3 eV, with a 500 Vpp collision RF. Quadrupole ion energy was 5.0 eV and low-mass cut-off was set to *m*/*z* 50. Focus pre-TOF transfer time was set to 45.0 µs and 8.0 µs for pre-pulse storage. MS/MS was turned on to enable isolation windows within quadrupole but not enabled in full-scan mode. Precursor *m*/*z* values were selected dependent on the drug or metabolite being isolated (e.g., *m*/*z* 195, 320, 152). Collision energy was set to 3 eV for transmission of ions without inducing fragmentation. MALDI MSI on brain tissue was performed on FMP10-sprayed slides immediately after matrix application. Imaging parameters included a mass scan range of *m*/*z* 50–1500 and a 50 µm laser raster width. Collison cell energy was set to 10 eV, with a 1200 Vpp collision RF. Quadrupole ion energy was 5.0 eV, and the low-mass cut-off was set to *m*/*z* 50. Focus pre-TOF transfer time was set to 70.0 µs, with a pre-pulse storage time of 7.5 µs. Precursor *m*/*z* values were selected based on the single- or double-derivatized and demethylated ions of HVA, 3-MT, and DOPAC (HVA/3-MT+[FMP-10]^+^, *m*/*z* 450.1699/435.2067; and DOPAC +2[FMP-10]-CH_3_^+^, *m*/*z* 689.2440). MS/MS was not activated in full-scan mode. MS/MS mode enabled quadrupole isolation widths varying from *m*/*z* 1, 2, 10, 30, and 100 for the liver tissue and dried droplet samples. For brain tissue, 1, 5, or 10 *m*/*z* isolation windows were used. Specific parameters are included within the figure captions.

### Data analysis

Data was analyzed using SCiLS Lab Pro (Bruker Corp, version 2025b) for all ion images and S/N calculations. Spectra were root mean square normalized for small-molecule drugs. Dopamine metabolite spectra were not normalized. Hotspot removal was performed and targeted analytes relative intensities were measured using a ± 5 mDa tolerance window. Drug analytes were assigned based on their [M+H]^+^ adducts. To readout peak intensities, the maximum peak intensity of the mean spectrum per region of interest was used. S/N ratios were calculated by dividing each analyte’s maximum peak intensity by the baseline noise of its mean spectrum per region from a matrix blank (Figure S1). Sensitivity improvement in S/N was calculated as the percent increase of quadrupole isolation (QI) S/N relative to full-scan S/N, defined as $(QI-fullscan)/full\;scan\times100$.

## Results and discussion

### Evaluation of limit of detection (LoD) and varying matrix applications on ITO slides

The 1 mmol/L drug stock solution of caffeine, chloroquine, and acetaminophen was serially diluted to establish the limit of detection (LoD) on the timsTOF fleX. In doing so, our goal was to establish a protocol and determine analyte response from the quadrupole isolations of our low-abundant small molecules.

**Fig. 1:**
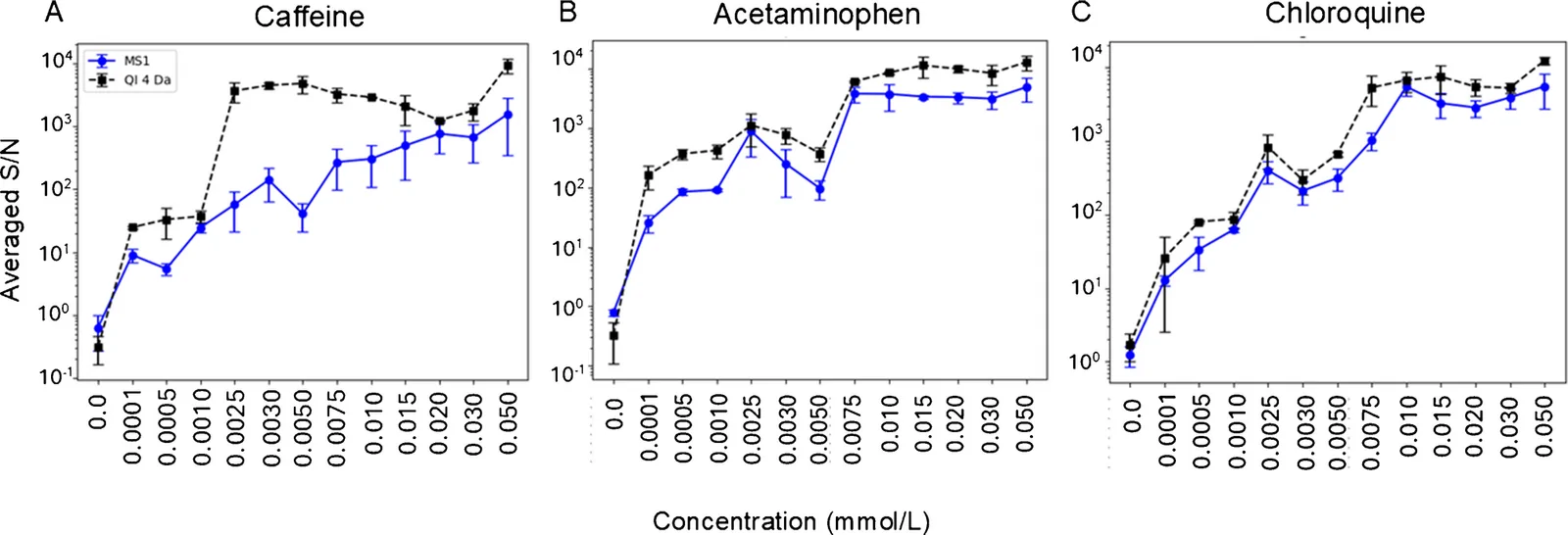
Determining the limit of detection in MALDI MSI of caffeine (**A**), acetaminophen (**B**), and chloroquine (**C**). One microliter of stock solution diluted to 0.0001 to 0.05 mmol/L per each drug, respectively, was spotted along with their associated dilutions and sprayed with DHB matrix. Blue solid lines are representative of normal full-scan (MS^1^) parameters without quadrupole isolation. Blue dashed lines are representative of MS/MS isolation windows set to 4.00 *m*/*z*, dependent on targeted analyte *m*/*z* value of interest (e.g., *m*/*z* 195 ± 4, 320 ± 4, 152 ± 4). S/N was analyzed using the (M+H)^+^ adduct for all targeted analytes. Point 0.00 mmol/L is representative of a matrix blank utilized for S/N calculations. *Y*-axis is normalized using log-based normalization

To improve reproducibility and spatial uniformity, we spotted standards on ITO slides and sprayed matrix using an automated matrix sprayer, which helped minimize variability in LoD measurements. Automated matrix application of a 1 µL of the drug mixture improved our spot-to-spot consistency relative to other methods, such as dried droplet (Figure S2). To evaluate sensitivity at low concentrations, the S/N was calculated and compared between the standard full-scan acquisition and a ± 2 *m*/*z* quadrupole isolation width across caffeine (Fig. 1A), acetaminophen (Fig. 1B), and chloroquine (Fig. 1C). At the lowest concentration (0.0001 mmol/L), caffeine, acetaminophen, and chloroquine had a 524 ± 43%, 188 ± 63%, and 156 ± 54% improvement, respectively. These S/N improvements were observed across all three compounds regardless of structure, class, or and *m*/*z* value. This application of quadrupole isolations improving S/N was also true for our electrospray ionization source (Figure S3), indicating that this S/N increase is not exclusive to a singular ionization source.

### Narrowing mass scan range versus utilizing quadrupole isolation widths for small-molecule drugs on liver tissue

**Fig. 2:**
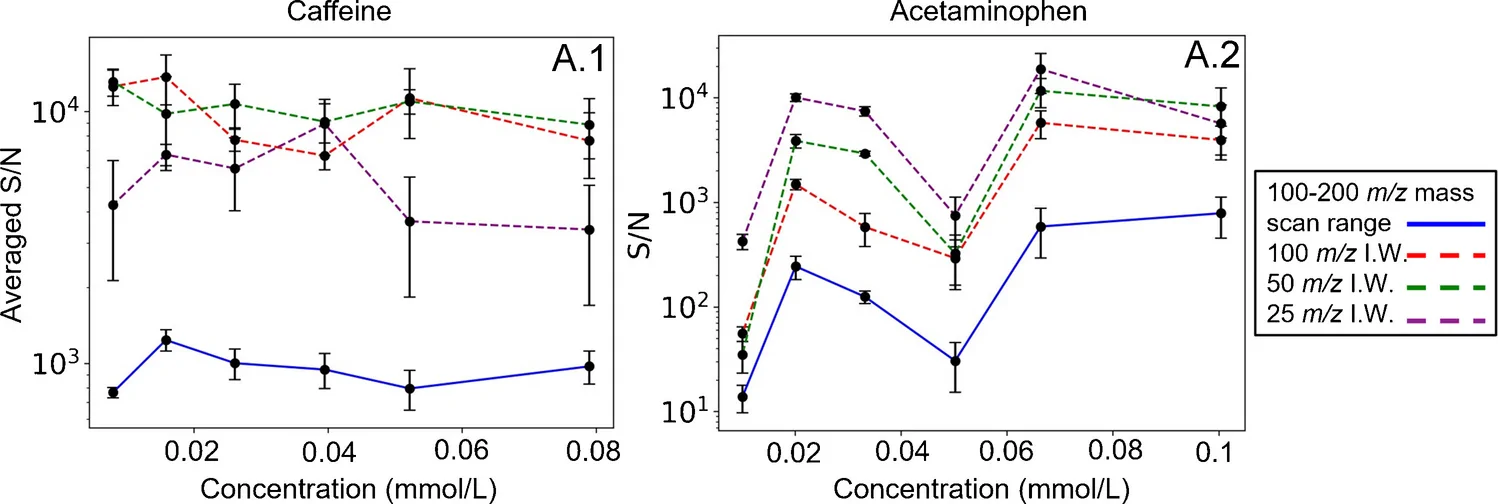
Comparing a narrow mass full-scan range (MS^1^) versus quadrupole isolation widths for caffeine (**A.1**) and acetaminophen (**A.2**). Narrow mass scan range (MS^1^) from *m*/*z* 100–200 is highlighted in solid blue lines while varying quadrupole isolation widths (100, 50, 25 *m*/*z*) are represented in dashed colored lines (100 *m*/*z*, red, 50 *m*/*z*, green, 25 *m*/*z*, violet). Three replicates of 1 µL spotted drug mixture, with associated dilutions, on liver tissue using a centered 100-pixel area were obtained to determine variance for each run performed utilizing the sprayed DHB matrix method. The [M+H]^+^ adduct was used for targeted analysis and for determining maximum peak intensities for correlating S/N calculations. Larger isolation widths (denoted as I.W.) used for this experiment in MS/MS mode were 25, 50, and 100 *m*/*z*. *Y*-axis is normalized using log-based normalization

The effects of varying the mass scan range and quadrupole isolation width on the signal-to-noise (S/N) ratio of selected drug analytes were systematically evaluated (Fig. 2). Caffeine (Fig. 2(A.1)) and acetaminophen (Fig. 2(A.2)) were chosen as representative compounds from a drug mixture (caffeine, acetaminophen, and chloroquine) applied to liver tissue.

A narrow scan range experiment was performed in MS^1^ mode without quadrupole isolation, employing a restricted acquisition window of *m*/*z* 100–200 to assess the impact of limiting the TOF mass range on S/N ratios. In contrast, isolation experiments were conducted in MS/MS mode, where the quadrupole was engaged to isolate targeted precursor ions using variable isolation widths of 25, 50, and 100 *m*/*z*.

Quadrupole isolation resulted in greater improvements in S/N compared to narrowing the mass scan range in MS^1^ mode. Targeted analysis of acetaminophen using quadrupole isolation widths of 100, 50, and 25 *m*/*z* resulted in average S/N increase of 1540 ± 91% compared to a narrowed mass scan range. This improvement was consistent across all concentration dilutions, with the lowest concentration (0.01 mmol/L) exhibiting a 4097 ± 30% S/N improvement using a 25 *m*/*z* isolation width. We note that acetaminophen (Fig. 2(A.2)) shows a steep decrease in S/N at 0.05 mmol/L across all replicates, which we attribute to a systematic variation in instrument performance or matrix crystallization. Targeted analysis of caffeine (Fig. 2(A.1)) showed averaged S/N increases of 1540 ± 60% for 100, 50, and 25 *m*/*z* isolation widths, respectively, relative to the narrow range full-scan acquisition. The lowest caffeine concentration (0.007 mmol/L) demonstrated a 665 ± 63% S/N increase with a 25 *m*/*z* isolation width.

Although both approaches reduce the total number of transmitted ions, quadrupole isolation more effectively enhances S/N by restricting ion transmission to the targeted *m*/*z* range prior to TOF analysis, thereby minimizing chemical noise arising from endogenous species present in the tissue. In contrast, narrowing the mass scan range often does not prevent off-target ions such as matrix clusters, background ions, and biological interferences from entering the TOF. As a result, ion suppression and background noise persist, leading to comparatively lower S/N improvements, as demonstrated for both caffeine and acetaminophen (Fig. 2). This effect is particularly relevant in MALDI MSI, where high chemical complexity and ion suppression often obscure target signals. However, it is important to note that quadrupole isolation acts downstream of ion generation and therefore cannot mitigate in-source effects such as competitive ionization or ion suppression, which occurs during desorption and ionization. Reducing the quadrupole isolation width can initially improve S/N by limiting co-transmitted interferences from matrix clusters and endogenous metabolites. However, the relationship between isolation width and S/N is not strictly linear. At very narrow isolation widths, reductions in chemical background primarily decrease the noise baseline. In contrast, moderate widening of the isolation window may allow partial transmission of additional background ions without a proportional increase in analyte signal, resulting in transient decreases in S/N. At wider isolation widths, enhanced transmission of the target analyte may compensate for the elevated baseline noise, ultimately yielding a net improvement in S/N. It is important to emphasize that quadrupole isolation occurs downstream of ion generation and therefore cannot mitigate in-source phenomena such as competitive ionization or ion suppression arising during desorption and ionization.

### Small quadrupole isolation widths effect on detecting low-abundant drugs in heterogeneous mixtures within dried droplet samples

**Fig. 3:**
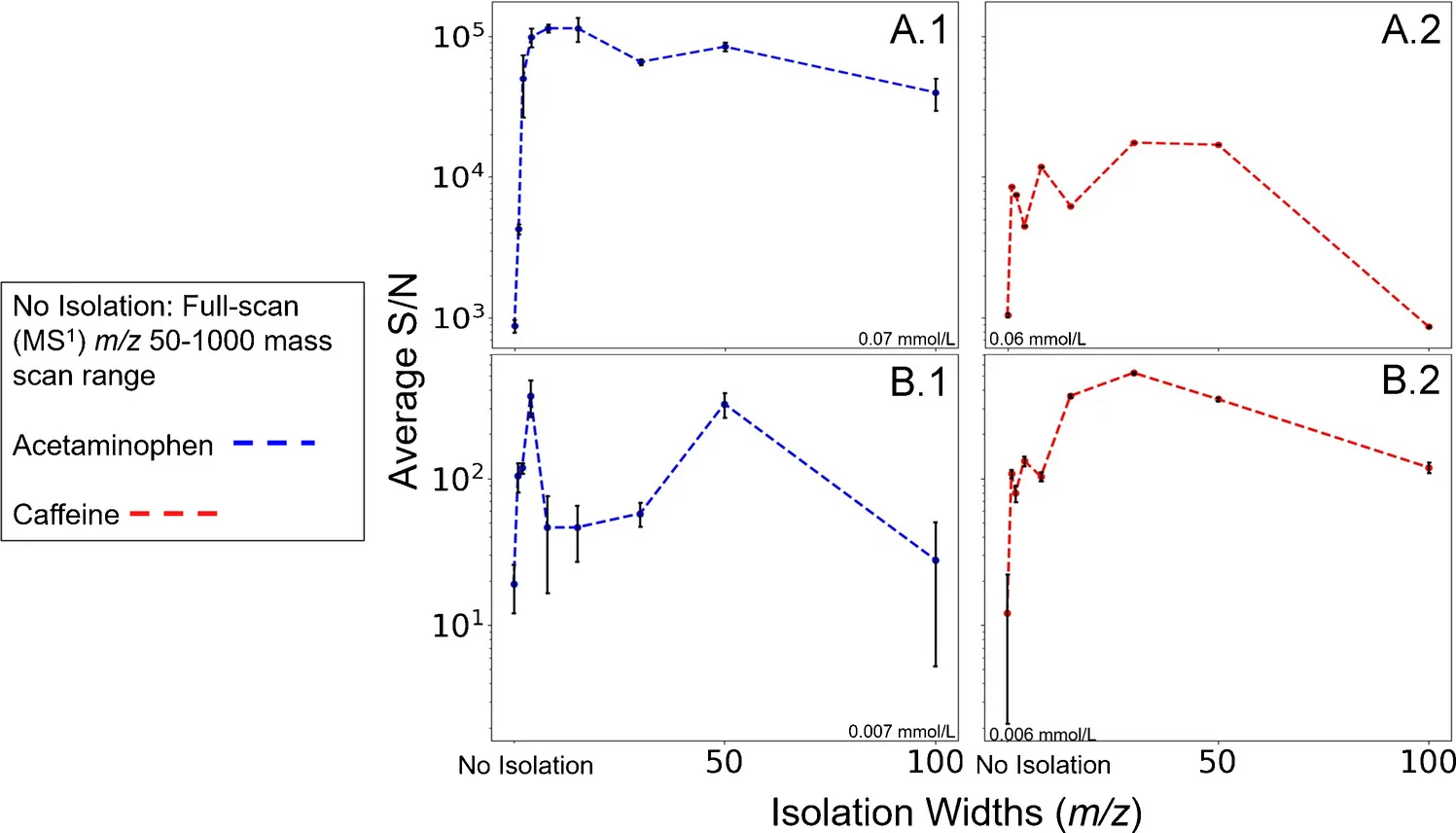
Comparing different quadrupole isolation widths on the S/N of acetaminophen and caffeine on tissue. Three 100-pixel regions of 1 µL dried droplets samples containing a mixture of chloroquine, caffeine, and acetaminophen. MS/MS mode was utilized for activating quadrupole isolation widths:1, 2, 4, 8, 15, 30, 50, 100 *m*/*z* for both a 0.07 and 0.06 mmol/L (**A.1**, **A.2**) and a 0.007 and 0.006 mmol/L (**B.1**, **B.2**) of both acetaminophen and caffeine, respectively. Full-scan normal MS^1^operation is represented with “No Isolation,” utilizing a mass scan range of *m*/*z* 50–1000. Peak intensity of both acetaminophen and caffeine were assessed using (M+H)^+^ adducts for both MS^1^ and MS/MS methods. For these experiments, the TOF mass scan range was maintained at a standard interval of *m*/*z* 50–1000 to isolate the effects of quadrupole transmission from those of scan range restriction. *Y*-axis is normalized using log-based normalization

Next, we investigated how quadrupole isolation widths influence overall S/N, using a mixture of chloroquine, acetaminophen, and caffeine. These samples were prepared directly on blank ITO slides to eliminate biological background and isolate the effects of quadrupole isolation on analyte signal. Dried droplet samples were analyzed at both high and low concentrations across a range of isolation widths (1, 2, 4, 8, 15, 30, 50, and 100 *m*/*z*) to assess whether lower analyte concentrations benefit from narrower isolation windows. Acetaminophen and caffeine were selected to determine whether the S/N enhancements observed at broader isolation widths (50 and 100 *m*/*z*) in the tissue-based experiments persisted in the absence of endogenous interferences (Fig. 3). Full-scan acquisition yielded the lowest S/N regardless of concentration, confirming that narrowing the MS^1^ mass scan range alone does not substantially improve S/N compared to quadrupole isolation.

To further assess this relationship, progressively narrower isolation windows were tested to determine whether increased selectivity improves S/N, especially at lower analyte concentrations where ion suppression and co-detected interferences are most significant. At higher concentrations, acetaminophen (0.07 mmol/L, Fig. 3(A.1)) exhibited the greatest S/N improvements at 1–4 *m*/*z* isolation widths, with increases of 344 ± 11%, 6792 ± 36%, and 10180 ± 10%, respectively, relative to full-scan acquisition.

Similarly, caffeine (0.06 mmol/L, Fig. 3(A.2)) showed maximal S/N enhancement at 1 and 8 *m*/*z*, improving by 629 ± 33% and 953 ± 25%, respectively. At lower concentrations (0.007 mmol/L for acetaminophen; 0.006 mmol/L for caffeine), the largest S/N gains were observed at isolation widths ≤ 15 *m*/*z*. Although spotting solutions were prepared at the stated concentrations, the effective sampled concentration was substantially lower, as only a fraction of the 1 µL droplet was ablated by the 50 µm laser spot size.

Under these conditions, acetaminophen exhibited an average S/N improvement of 372 ± 23%, while caffeine improved by 886 ± 152% for the lowest concentration and 1 *m*/*z* width. For both analytes, S/N decreased at broader isolation widths (50–100 *m*/*z*), with a more pronounced decline at lower concentrations. No consistent linear relationship between isolation width and concentration was observed; however, narrower windows consistently yielded higher S/N, with 1 *m*/*z* isolation producing the most pronounced gains relative to MS^1^ acquisitions. Despite reduced absolute signal intensity under the narrowest conditions (1 and 2 *m*/*z* QI), substantial baseline noise reduction resulted in net S/N improvements of relatively 472% for acetaminophen at low concentration. A similar effect was observed for caffeine (with 1 and 2 *m*/*z* QI) where partial overlap with matrix ions (e.g., *m*/*z* 195.0256) persisted, yet S/N improvements of relatively 818% were still achieved.

Despite overall improvements in S/N, interference was observed at the narrowest isolation widths (1–10 *m*/*z*). DHB generated background peaks at *m*/*z* 154 and 155, near the targeted [acetaminophen+H]^+^ ion (*m*/*z* 152.07). Even under narrow isolation conditions, a substantial ion population was transmitted to the TOF, and the relatively intense matrix ions contributed to a residual signal within the isolation window. Although matrix-derived ions were present in both isolated and full MS^1^ spectra, their relative contribution became more apparent under narrow isolation conditions due to the reduced total ion flux entering the TOF. Nevertheless, 1 and 2 *m*/*z* isolation widths still produced higher S/N ratios than full-scan acquisition, reflecting the benefits of reduced chemical noise and improved spectral clarity. The lowest baseline noise was achieved with 1 *m*/*z* isolation (Figure S4). However, narrow isolation windows resulted in up to a ten-fold reduction in analyte intensity compared to broader windows (Figure S4), highlighting the trade-off between selectivity and sensitivity. In cases of spectral competition, the most abundant ion within the isolation window may dominate transmission, potentially biasing quantitative measurements. This balance between improved noise suppression and reduced ion flux was consistently observed across narrow isolation widths (2–10 *m*/*z*) and was particularly advantageous for detecting low-abundance small-molecule drugs.

### Using narrow isolation windows to improve S/N of small-molecule drugs and their associated metabolites on tissue

**Fig. 4:**
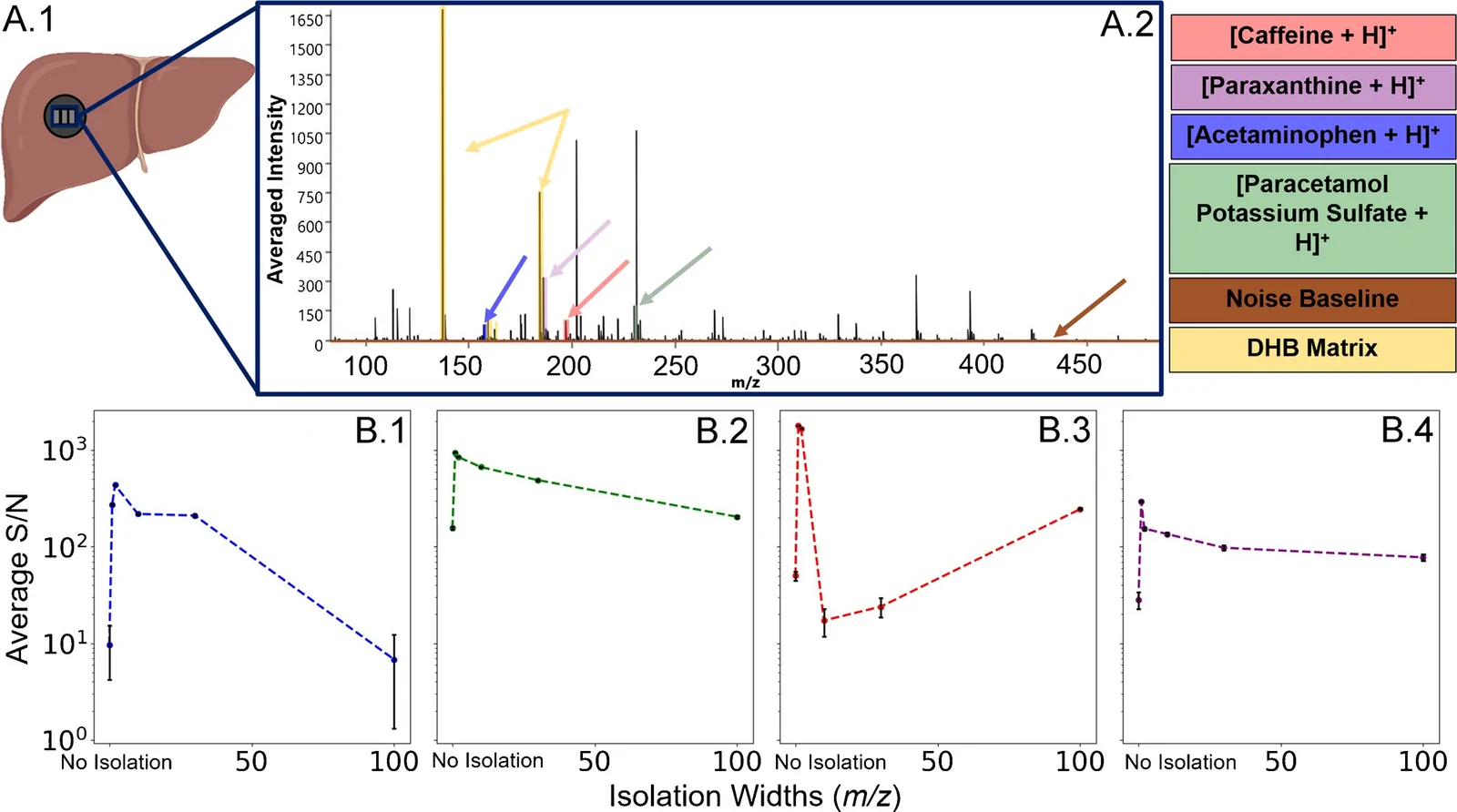
Utilizing MS/MS isolation windows on small-molecule drugs and their associated metabolites on liver tissue. MALDI MSI MS^1^ acquisition is denoted as “No Isolation” and MS/MS isolation acquisition is denoted on *X*-axis, ranging from 1 to 100 *m*/*z*. The spotted solution contained a mixture of caffeine, paraxanthine, acetaminophen, and paracetamol sulfate spotted on liver tissue and sprayed with DHB matrix (**A.1**). Three 100-pixel areas within center of each droplet were assigned for MALDI MSI acquisition (**A.1**) represented by grey boxes. The averaged spectra (**A.2**) were taken from the three acquisition areas to determine the S/N of the targeted drugs and their metabolites from normal full-scan MSI runs and MS/MS quadrupole isolation widths. The average S/N was calculated for the [M+H]^+^ adducts of acetaminophen (0.06 mmol/L, **B.1**), paracetamol potassium sulfate (0.03 mmol/L, **B.2**), caffeine (0.06 mmol/L, **B.3**), and paraxanthine (0.05 mmol/L, **B.4**). The noise baseline is highlighted (orange) for emphasis in S/N calculations, and associated DHB matrix peaks are highlighted (yellow) to emphasize signal comparison to targeted molecules. *Y*-axis is normalized using log-based normalization

To further evaluate the effects of quadrupole isolation observed in dried droplet mixtures, we investigated whether S/N improvements could be achieved in the presence of complex endogenous biological background. Specifically, S/N behavior was assessed for the parent drugs caffeine and acetaminophen and their primary metabolites, 1,7-dimethylxanthine (paraxanthine) and paracetamol sulfate, respectively. We evaluated the quadrupole S/N improvement effect of the mixture spotted onto liver tissue and sprayed DHB matrix (Fig. 4(A.1)). This experimental design allowed us to evaluate both parent compounds and metabolites in a more physiologically relevant and complex environment, where matrix effects and endogenous ions often suppress analyte signals. A range of quadrupole isolation widths, from narrow (1–15 *m*/*z*) to broad (25–100 *m*/*z*), were tested to determine selectivity influenced S/N on tissue.

Under narrow isolation widths, all four analytes showed improvements in S/N relative to MS^1^ acquisition (*m*/*z* 50–600), with increases consistently exceeding 80%. Acetaminophen exhibited an 3156 ± 58% improvement in S/N at a 1 *m*/*z* isolation window (Fig. 4(B.1)), while its metabolite, paracetamol sulfate, showed an 531 ± 42% S/N gain under the same conditions (Fig. 4(B.2)). As expected, broader isolation widths (e.g., 100 *m*/*z*) led to decreased S/N values due to increased chemical noise and matrix ion overlap from various salt adducts (e.g., Na^+^). Caffeine depicted a 4189 ± 8% S/N enhancement at 1 *m*/*z* window in the lowest concentration condition (Fig. 4(B.3)), with a sharp decline in S/N beyond the 2 *m*/*z* window. Interestingly, caffeine displayed a resurgence in S/N at the broadest window tested (100 *m*/*z*). This was a pattern consistent with the quadrupoles ability to increase S/N previously seen in the dried droplet experiments. Despite paraxanthine being structural similarity to caffeine, it behaved more like paracetamol sulfate in response to QI. Paraxanthine showed greatest S/N improvements of 797 ± 15% at 1 *m*/*z* isolation width (Fig. 4(B.4)). While narrow windows enhanced detection sensitivity by suppressing background interference, the magnitude and pattern of S/N response varied between analytes. This divergence in isolation width response between structurally related analytes such as caffeine and paraxanthine suggests that chemical properties like ionization efficiency and gas-phase stability play a more critical role than structural similarity alone.

In contrast to fragmentation-based detection, quadrupole isolation preserves the intact precursor ion, avoiding multiple product ions. For small molecules such as acetaminophen and caffeine, MS/MS often produces low *m*/*z* fragments with limited specificity that fall within a chemically crowded region of the mass spectrum, thereby increasing baseline noise and reducing S/N for individual product ions. To directly assess this effect, MS/MS experiments were performed and compared to quadrupole isolation across concentrations (Figure S5). MS/MS experiments were conducted using 20 eV CID and monitoring the dominant fragments (*m*/*z* 110 for acetaminophen and *m*/*z* 138 for caffeine). These findings support the use of quadrupole isolation over MS/MS for small-molecule imaging in complex tissue environments.

#### Applying quadrupole isolation windows method for increasing S/N of dopamine metabolite standards spotted on liver tissue

Previous results demonstrated that applying narrow quadrupole isolation widths significantly enhances S/N for small-molecule drugs and their metabolites in complex biological matrices, such as liver tissue. Building on these findings, we evaluated whether this strategy could similarly improve S/N for endogenous metabolites, specifically dopamine metabolites, in brain tissue. Dopamine serves as a key biomarker of nigrostriatal integrity and plays a crucial role in studying neurodegenerative disorders such as Parkinson’s disease, where dopamine depletion represents a defining pathological hallmark [52, 53]. Its major metabolites, homovanillic acid (HVA), 3-methoxytyramine (3-MT), and dihydroxyphenylacetic acid (DOPAC), provide valuable biochemical readouts of dopamine turnover and are widely used as biomarkers in both research and clinical settings. Assessing these metabolites has the advantage of reflecting activity across both principal dopamine catabolic pathways [54]. Importantly, pharmacological interventions can differentially modulate these pathways, a shift that would remain undetected when relying solely on dopamine levels. While dopamine itself is readily detectable in full-scan MS1 after FMP-10 derivatization [55, 56], its low abundance metabolites, especially HVA, approach the limit of detection even post-derivatization. The difficulty increases in brain tissue, where background ions and matrix effects further reduce analyte signals. Thus, enhancing S/N for dopamine metabolites is critical for achieving robust, reproducible detection and enabling their confident use in physiological and pathophysiological studies.

**Fig. 5:**
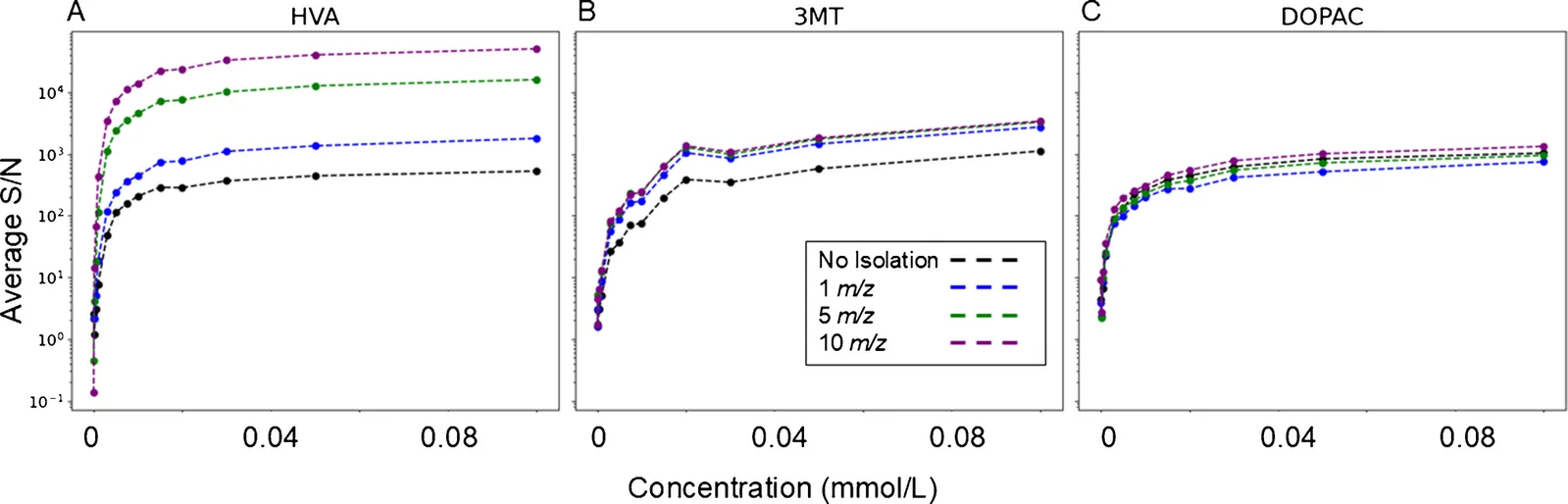
Evaluation of S/N improvements for dopamine metabolites, HVA (**A**), 3-MT (**B**), and DOPAC (**C**), utilizing dry droplet method with sprayed matrix. Quadrupole isolation widths of 1 *m*/*z* (blue), 5 *m*/*z* (green), and 10 *m*/*z* (purple) were compared to full-scan acquisition with no isolation (black). The plots illustrate changes in S/N as a function of isolation width for each metabolite. Corresponding mass spectra are provided in the Supporting Information 3. Point 0.00 mmol/L is representative of a matrix blank utilized for S/N calculations. *Y*-axis is normalized using log-based normalization

To address these challenges, we evaluated the quadrupole isolation widths method (10, 5, and 1 *m*/*z*) on the detection of HVA, 3-MT, and DOPAC, and compared these results to full-scan measurements with no isolation (Fig. 5). Initial experiments were performed by spotting 0.5 µL of a mixed metabolite stock solution onto liver tissue followed by FMP-10 matrix application. Detection proceeded through formation of the (M+FMP-10)⁺ adducts, with the applied isolation width indicated in each figure panel. For HVA (Fig. 5A), the 10 *m*/*z* isolation width produced the most substantial improvement in S/N, yielding an average increase of 6571 ± 2508% relative to no isolation. A 5 *m*/*z* isolation width also generated a pronounced enhancement, with an average improvement of 1989 ± 822%. In contrast, the 1 *m*/*z* width produced only a modest increase (146 ± 48%). These results indicate that broader isolation widths (10 and 5 *m*/*z*) are markedly more effective for improving HVA detection. For 3-MT (Fig. 5B), both the 10 *m*/*z* and 5 *m*/*z* isolation widths provided comparable gains in S/N, with average increases of 187 ± 65% and 177 ± 61%, respectively. The 1 *m*/*z* width offered a smaller, though still measurable, enhancement (115 ± 47%). These findings suggest that 10 and 5 *m*/*z* isolation widths are optimal for boosting 3-MT signal intensity, while narrower isolation provides only moderate improvement. For both HVA and 3-MT, we noted that the percent S/N improvements appear substantially larger than those observed for the small-molecule drug data. This effect arises from the extremely low baseline S/N of these endogenous metabolites under full-scan conditions. Because they are difficult to detect without isolation, their S/N values approach 1; consequently, even moderate absolute increases in S/N translate into disproportionately large percentage increases. Thus, while the calculated percent improvements exceed 5000% in some cases, these values primarily reflect the very low starting S/N rather than unusually strong absolute enhancements.

For DOPAC (Fig. 5C), the 10 *m*/*z* isolation width again resulted in the greatest S/N enhancement, with an average increase of 35 ± 22% compared to no isolation. Interestingly, the condition with no isolation produced the second-highest S/N, indicating that DOPAC may be sufficiently detected without mass filtering. In contrast, the 5 *m*/*z* isolation width showed no improvement (− 18 ± 19%), and the 1 *m*/*z* width resulted in a slight decrease in S/N (− 2 ± 18%). These results suggest that only the broadest isolation width (10 *m*/*z*) meaningfully benefits DOPAC detection, while narrower widths may suppress the signal. Because the chemical noise profile of FMP-10 differs from that of non-derivatized samples analyzed with DHB, the effect of isolation width is also matrix-dependent. Overall, these findings demonstrate that quadrupole isolation width exerts a strong and analyte-dependent influence on S/N performance. While broader isolation consistently enhances detection of HVA and 3-MT, only DOPAC shows minimal benefit, underscoring the importance of tailoring isolation strategies to the specific chemical properties and ionization behavior of each metabolite. We also note that while OTCD is common for the detection of these metabolites, it is not applicable to all small molecules.

#### Applying quadrupole isolation windows method for increasing S/N of dopamine metabolites in rat brain tissue

**Fig. 6:**
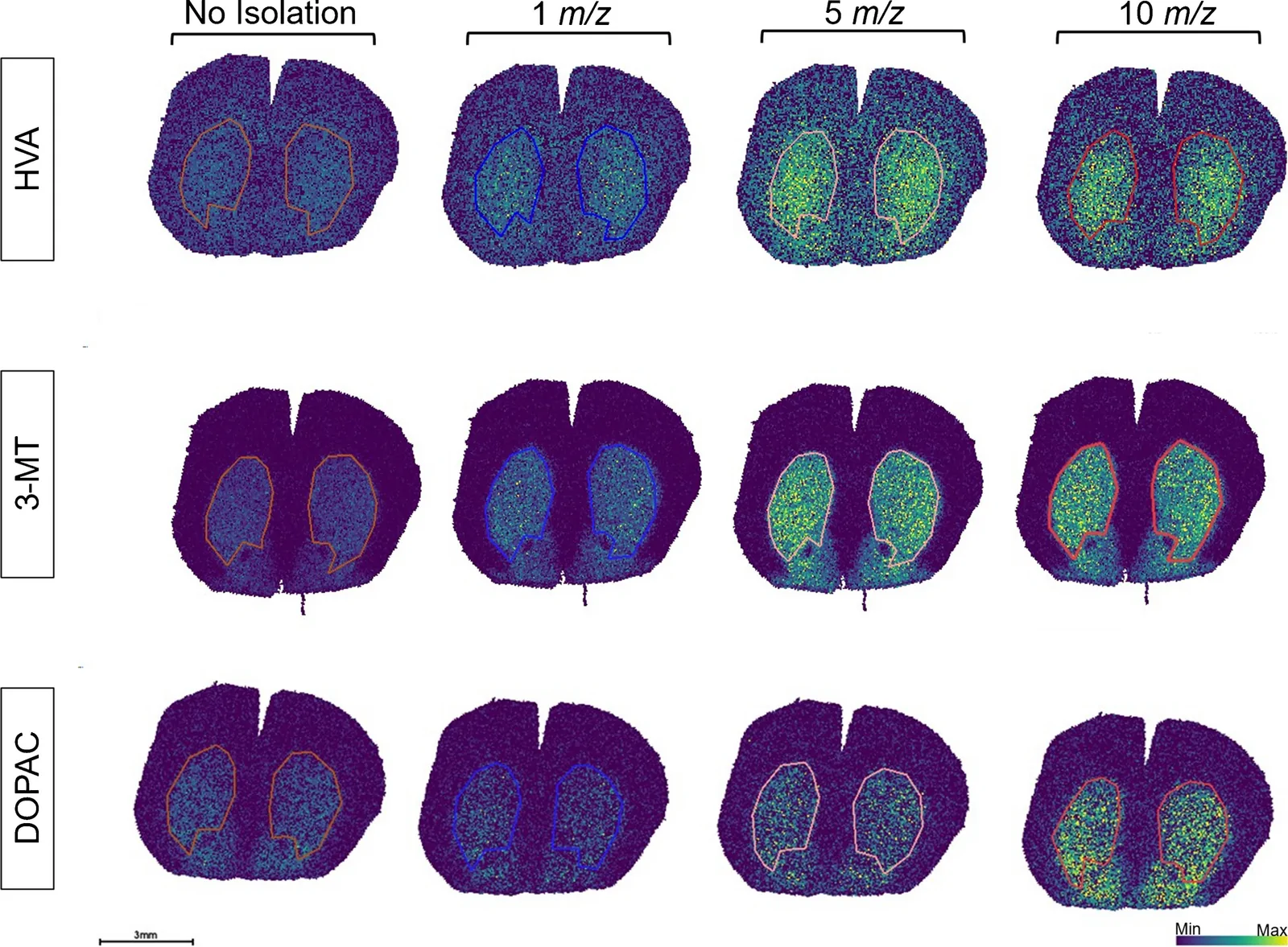
Ion images of dopamine metabolites (HVA, 3-MT, and DOPAC) acquired using varying quadrupole isolation widths in coronal rat brain tissue. Metabolite localization is observed primarily within the striatal ROI at bregma levels 0.60–0.24 mm. Each isolation condition is depicted using a distinct color scheme: no isolation (orange), 1 *m*/*z* (blue), 5 *m*/*z* (pink), and 10 *m*/*z* (red). Corresponding spectral references are provided in Supporting Information 4

Building on the results obtained from spotted standards on liver tissue, we next assessed the influence of quadrupole isolation widths directly in rat brain tissue. This analysis aimed to determine whether the trends observed in the liver-based standard experiments could be reproduced in a more biologically relevant matrix. As before, we examined the effects of different isolation widths on the detection of endogenous HVA, 3-MT, and DOPAC. Figure 6 presents the spatial distribution of HVA within the striatum, accompanied by the corresponding mass spectrum, and illustrates the impact of each isolation width on signal quality.

For HVA, the 10 *m*/*z* isolation width again yielded the most pronounced improvement in S/N, producing a 4892% increase relative to no isolation. Consistent with the spotted standard results, this broader isolation width provided the greatest enhancement in detection sensitivity. The 5 *m*/*z* width also produced a substantial increase in S/N (1526%), whereas the 1 *m*/*z* width resulted in only a modest improvement (86%). Overall, these findings mirror the trends observed in the liver standard experiments and further support the utility of broader quadrupole isolation widths for enhancing endogenous metabolite detection in complex tissue environments.

For 3-MT, the 10 *m*/*z* and 5 *m*/*z* isolation widths produced nearly identical improvements in S/N, yielding increases of 228% and 198%, respectively, relative to no isolation. These results indicate that both isolation widths are comparably effective for enhancing 3-MT detection. The 1 *m*/*z* width produced a smaller, yet still meaningful, improvement of 110%. Collectively, these findings suggest that 10 *m*/*z* and 5 *m*/*z* isolation widths are optimal for improving detection sensitivity, while 1 *m*/*z* provides only moderate enhancement. This trend is consistent with the results obtained from the spotted standards on liver tissue. For DOPAC, the 10 *m*/*z* isolation width produced the most substantial improvement in S/N, with an 82% increase compared to no isolation. Notably, the full-scan (no isolation) condition yielded the second-highest signal, suggesting that DOPAC may be efficiently detected even without mass filtering. In contrast, the 5 *m*/*z* isolation width produced no enhancement (− 6%), and the 1 *m*/*z* width resulted in only a modest increase (25%). These observations mirror the trends observed within standard experiments. These results indicate that, for DOPAC, a 10 *m*/*z* isolation width is most effective for improving S/N, whereas narrower widths provide minimal benefit or may even diminish signal quality.

Applied quadrupole isolations narrowed the instrument’s effective scanning range by restricting acquisition to a targeted *m*/*z* window but generated specific ion packets to the TOF analyzer that increased S/N of targeted analytes. We defined this increase of S/N to be ≥ 50% as the threshold for meaningful improvement. Neurotransmitters were best analyzed using raw, unnormalized data and showed maximal S/N improvement at a broad 10 *m*/*z* isolation width. In contrast, small-molecule drugs benefited from RMS normalization and performed best with a narrow 1 *m*/*z* window. These differences likely reflect distinct ionization behavior and isotopic or adduct complexity between endogenous metabolites and exogenous drug standards. Together, these results show that quadrupole isolation parameters must be tailored to the specific analyte class. This work provides practical guidance for optimizing isolation strategies in MALDI analyses and establishes a foundation for expanding these approaches to other endogenous metabolites and low abundance exogenous materials.

## Conclusion

Isolation widths of 10 *m*/*z*, dependent on *m*/*z* of analyte, and below exhibited an improvement in S/N and lowered spectral noise. This improvement in S/N was consistent for the drugs of interest, their associated metabolites, and neurotransmitters. With a broader window (10 *m*/*z*), the quadrupole may transmit ions more effectively, including ions at exact mass, due to reduced filtering stringency. Narrower windows (1 *m*/*z*) require more precise filtering, which can lead to losses in transmission efficiency for the targeted mass. These findings emphasize the importance of fine-tuning quadrupole isolation widths to enhance the detection of specific analytes in complex biological samples. While broader isolation widths appear to be more effective for dopamine metabolites, this was not the case for the small-molecule drugs. This suggests that optimization may need to be adapted per individual analyte. Future studies should investigate the mechanisms underlying these differences in performance and assess whether similar trends apply to other larger drug molecules and neurotransmitters within biological matrices. Exploring these aspects further might shed light on how quadrupole isolation widths, analyte characteristics, and instrument design work together to influence detection outcomes.

## Supplementary Information

Below is the link to the electronic supplementary material.

Supplementary file1 (DOCX 5.66 MB)

## Data Availability

The data will be provided by the authors upon reasonable request.
