# Supplementary material for: Improving detection of low-abundant pharmaceuticals and neurotransmitters via quadrupole isolation in MALDI mass spectrometry imaging
Source: Anal Bioanal Chem. 2026 Jul 14;418(20):6749–61. doi: 10.1007/s00216-026-06659-z (PMC13619658; doi:10.1007/s00216-026-06659-z)
Supplement: Supplementary file 1 [file 216_2026_6659_MOESM1_ESM.docx]

**Supporting Information**

**Improving detection of low abundant pharmaceuticals and neurotransmitters via quadrupole isolation in MALDI Mass Spectrometry Imaging**

Karina A. Vargas^1^, Sarah Diez^2^, Michael Becker^2^, Elizabeth K. Neumann^1^

1. Department of Chemistry, University of California, Davis

2. Boehringer Ingelheim Pharma GmbH, Biberach an der Riss, Germany

**Table of Contents**

1. Figure S1. S/N calculations

2. Figure S2. Coffee ring effect versus centered-droplet acquisition

3. Figure S3. ESI orthogonal verification of acetaminophen, caffeine, and chloroquine S/N improvements

4. Figure S4. Raw spectra of effect of varying quadrupole isolation widths on MALDI spectra of caffeine and acetaminophen.

5. Figure S5. MS/MS of acetaminophen and caffeine for S/N comparison.

6. Figure S6. Raw spectra and ion images of effect of quadrupole isolation width HVA, 3-MT, and 3,4- DOPAC

7. Figure S7. Raw spectra of selected analyte *m/z* of effect of quadrupole isolation width on endogenous HVA, 3-MT, and DOPAC in rat brain tissue

Figure S1. Example S/N calculation using caffeine (*m/z* 195). S/N was calculated by dividing the peak height of the targeted analyte by the lowest discernible noise baseline measured from the blank DHB spectra in SCiLS Lab within a ±0.05 Da window centered on the analyte *m/z*.

Figure S2. Illustration of the coffee-ring effect versus centered droplet acquisition. To mitigate the coffee-ring effect intrinsic to MALDI droplet imaging, three 100-pixel regions centered within each 1 µL standard droplet (with acetaminophen, chloroquine, and caffeine) were selected for analysis, minimizing edge-related enrichment and reducing variability from non-uniform analyte distribution.

Figure S3: ESI replicates of lower concentrations for LoD and observance of S/N increase. MRM parameters included: *m/z* 5 isolation width, 5 eV collision energy, 15 eV IsCID, and 5 μL/min flow rate. Acetaminophen (A) caffeine (B) and chloroquine (C) were analyzed within a stock mixture of 50/50 MeOH:H_2_O. Blue solid lines are full scan parameters with no MRM protocol to observe S/N increase. Y-axis is normalized using log base normalization.

Figure S4: Effect of quadrupole isolation width on MALDI spectra of caffeine (A) and acetaminophen (B). Narrow isolation windows (1 Da, A.1 and B.1) selectively transmit ions of interest, resulting in cleaner spectra with reduced matrix interference and improved signal-to-noise. Broader isolation windows (100 Da, A.2 and B.2) allow more co-isolated ions, producing noisier spectra with lower resolution of the target analyte peaks. Additionally, y-axis served to depict absolute intensity values that decreased in orders of magnitude for smaller isolation widths (A.1, B.1) compared to larger (A.2, B.2).

Figure S5. Comparison of MS/MS and quadrupole isolation approaches for the analysis of acetaminophen and caffeine. Quadrupole isolation was performed on the protonated parent ions of acetaminophen and caffeine using a 4 Da isolation window. For MS/MS experiments, CID was applied at 20 eV with a 1 Da acquisition window. The primary product ions monitored were *m/z* 110 for acetaminophen and *m/z* 138 for caffeine. The far-right column represents the percent improvement in S/N for QI relative to MS/MS, demonstrating that quadrupole isolation of the intact precursor ion provided greater S/N compared to fragmentation-based detection.

| **Acetaminophen** | | | |
| --- | --- | --- | --- |
| **Concentration (mM)** | **Quadrupole Isolation of Parent Ion S/N (*m/z* 152)** | **MSMS of Main Fragment Ion S/N (*m/z* 110)** | **% Improvement of QI vs MSMS** |
| 0.05 | 9420.66 ± 30 | 151.58 ± 36 | 6620 |
| 0.03 | 1788.46 ± 68 | 99.27 ± 47 | 1895 |
| 0.02 | 1245.10 ± 28 | 99.48 ± 27 | 1219 |
| 0.015 | 2089.76 ± 12 | 11.70 ± 3 | 20124 |
| 0.01 | 2916.66 ± 34 | 17.68 ± 21 | 44557 |
| 0.0075 | 3280.63 ± 15 | 70.95 ± 28 | 4894 |
|  |  |  |  |
|  |  |  |  |
| **Caffeine** | | | |
| **Concentration (mM)** | **Quadrupole Isolation of Parent Ion S/N (*m/z* 195)** | **MSMS of Main Fragment Ion S/N (*m/z* 138)** | **% Improvement of QI vs MSMS** |
| 0.05 | 12842.76 ± 43 | 426.69 ± 48 | 3139 |
| 0.03 | 8359.1 ± 37 | 344.16 ± 21 | 2970 |
| 0.02 | 9859.22 ± 13 | 303.96 ± 21 | 3596 |
| 0.015 | 11290.47 ± 52 | 46.26 ± 14 | 26641 |
| 0.01 | 8528.57 ± 48 | 66.72 ± 18 | 13302 |
| 0.0075 | 6079.68 ± 18 | 274.69 ± 62 | 2213 |

Figure S6: Effect of quadrupole isolation width on MALDI-MSI spectra of homovanillic acid (HVA), 3-methoxytyramine (3-MT), and 3,4-dihydroxyphenylacetic acid (DOPAC) standard solutions (0–100 µM) spotted on liver tissue, with corresponding ion images. Increased isolation widths consistently yielded higher signal intensities across all dopamine metabolites.

Figure S7: Effect of quadrupole isolation width on MALDI-MSI spectra of endogenous homovanillic acid (HVA), 3-methoxytyramine (3-MT), and 3,4-dihydroxyphenylacetic acid (DOPAC) in rat brain tissue. Broader isolation widths (5 and 10 Da) clearly resulted in higher signal intensities across all dopamine metabolites.
